# Risk stratification of elderly patients with Brugada syndrome: Results from a large Japanese cohort of idiopathic ventricular fibrillation

**DOI:** 10.1002/joa3.70047

**Published:** 2025-03-24

**Authors:** Tetsuji Shinohara, Masahiko Takagi, Tsukasa Kamakura, Yuki Komatsu, Yoshiyasu Aizawa, Yukio Sekiguchi, Yasuhiro Yokoyama, Naohiko Aihara, Masayasu Hiraoka, Kazutaka Aonuma, for the Japan Idiopathic Ventricular Fibrillation Study (J‐IVFS) Investigators

**Affiliations:** ^1^ Department of Cardiology and Clinical Examination, Faculty of Medicine Oita University Oita Japan; ^2^ Department of Medicine II Kansai Medical University Moriguchi Japan; ^3^ Division of Arrhythmia and Electrophysiology, Department of Cardiovascular Medicine National Cerebral and Cardiovascular Center Osaka Japan; ^4^ Department of Cardiology, Faculty of Medicine University of Tsukuba Tsukuba Japan; ^5^ Department of Cardiovascular Medicine Nippon Medical School Tokyo Japan; ^6^ Department of Cardiovascular Internal Medicine Sakakibara Heart Institute Fuchu Japan; ^7^ Division of Cardiology, Department of Internal Medicine Daisan Kitashinagawa Hospital Tokyo Japan; ^8^ Department of Internal Medicine Senri Central Hospital Suita Japan; ^9^ Department of Cardiology Tokyo Medical and Dental University Tokyo Japan; ^10^ Department of Cardiology Saiseikai Mito Hospital Mito Japan

**Keywords:** Brugada syndrome, elderly, programmed electrical stimulation, risk stratification, ventricular fibrillation

## Abstract

**Background:**

Brugada syndrome (BrS) is an inherited cardiac channelopathy associated with a high risk of sudden cardiac death (SCD) due to ventricular fibrillation (VF). Although implantable cardioverter‐defibrillators (ICDs) are the primary therapy for SCD prevention, the risk stratification of elderly patients with BrS remains unclear. This study aimed to evaluate the incidence and risk factors of life‐threatening arrhythmias in elderly patients with BrS.

**Methods:**

We analyzed 523 patients with BrS (mean age 51 ± 13 years, 497 men) enrolled in the multicenter prospective Japan Idiopathic Ventricular Fibrillation Study. Patients were categorized into the elderly (>60 years, *n* = 150) and nonelderly (≤60 years, *n* = 373) groups. Clinical characteristics, programmed electrical stimulation (PES) results, and outcomes, including cardiac events (CEs: VF, fast ventricular tachycardia, or SCD), were compared. Statistical analyses were performed using Kaplan–Meier curves and Cox proportional hazard models.

**Results:**

During a mean follow‐up of 106 ± 62 months, 59 patients (11%) experienced CE. The annual CE incidence was lower in the elderly group than in the nonelderly group (0.7% vs. 1.5%, *p* = 0.016). History of VF independently predicted CE occurrence in elderly patients (hazard ratio: 23.5, *p* < 0.001). Asymptomatic elderly patients exhibited a negligible risk of CE. PES did not predict CE occurrence in the elderly group.

**Conclusions:**

Elderly patients with BrS have a better prognosis than nonelderly patients, particularly if they are asymptomatic. A history of VF is a key risk factor for life‐threatening arrhythmias in elderly patients with BrS.

## Graphical abstract

This study suggests that asymptomatic elderly patients with Brugada syndrome may be at a very low risk of developing life‐threatening arrhythmias.

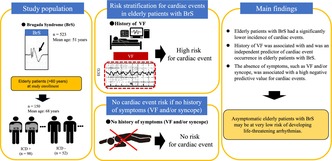

## 1 INTRODUCTION

Brugada syndrome (BrS) is an inherited cardiac channelopathy that is diagnosed by the presence of coved‐type ST segment elevation on electrocardiogram (ECG) leads *V*_1_ and *V*_2_ in structurally normal hearts and is associated with a high risk of sudden cardiac death (SCD) due to life‐threatening arrhythmias.^1^ The first cardiac event (CE), including fast polymorphic ventricular tachycardia (VT) or ventricular fibrillation (VF), generally occurs in patients aged 40–50 years but rarely in the elderly.^2^, ^3^, ^4^ Minier et al.^5^ divided patients with BrS into three groups according to age (under 17 years, 17–60 years, and over 60 years) to investigate differences in prognosis among patients with BrS in each age group. They reported that the oldest patients present the lowest risk of SCD. Implantable cardioverter‐defibrillator (ICD) is considered to be the main therapy for SCD prevention in patients with BrS. However, several reports have suggested a high prevalence of long‐term complications, such as inappropriate shock, lead failure, and device infection.^4^, ^6^ In a single‐center study, Kamakura et al.^7^ conducted a long‐term follow‐up of high‐risk patients with BrS with ICDs and reported a low incidence of VF in patients >70 years old. Therefore, some older patients with BrS are at an extremely low risk of developing VF and may not necessarily require ICD implantation or replacement. Although several large clinical studies have proposed risk factors for future VF,^1^, ^8^ none have detailed the incidence of VF or the need for an ICD in elderly patients with BrS. Moreover, case reports on arrhythmic events in elderly patients with BrS are few.^9^, ^10^ Nevertheless, despite the small number of cases, the risk of SCD in patients with BrS should never be ignored. In fact, ICD implantation or replacement is recommended for patients with BrS who are at high risk of VF occurrence, including the elderly. To the best of our knowledge, no large multicenter prospective clinical study has been conducted on the risk stratification of elderly patients with BrS. Considering age‐related conduction disturbances and hormonal changes,^11^, ^12^ the risk factors of BrS may differ between elderly and middle‐aged patients.

The purpose of this study was to determine the incidence and risk factors for life‐threatening arrhythmias in elderly patients with BrS. The clinical characteristics of elderly patients with BrS were evaluated using the database of the multicenter prospective Japan Idiopathic Ventricular Fibrillation Study (J‐IVFS) on patients with idiopathic VF, including BrS.

## 2 METHODS

### 2.1 Study population

Consecutive patients diagnosed with type 1 BrS ECG between February 2002 and January 2015 according to the proposed BrS diagnostic criteria were enrolled in the J‐IVFS.^1^ The present study included a total of 523 patients (mean age, 51 ± 13 years, 497 men), who comprised the same cohort described in a previous publication.^13^ The patients were probands from 523 families who were followed‐up for >1 year and met the following inclusion criteria: (1) normal findings on physical examination, chest radiography, and echocardiography; (2) no intake of antiarrhythmic drugs; and (3) absence of electrolyte abnormalities at the time of ECG recording and other examinations. The current study had the largest survey of patients with BrS in Japan. The cohort was selected using the same BrS inclusion criteria as those used in other European studies.^2^, ^14^

**FIGURE 1:**
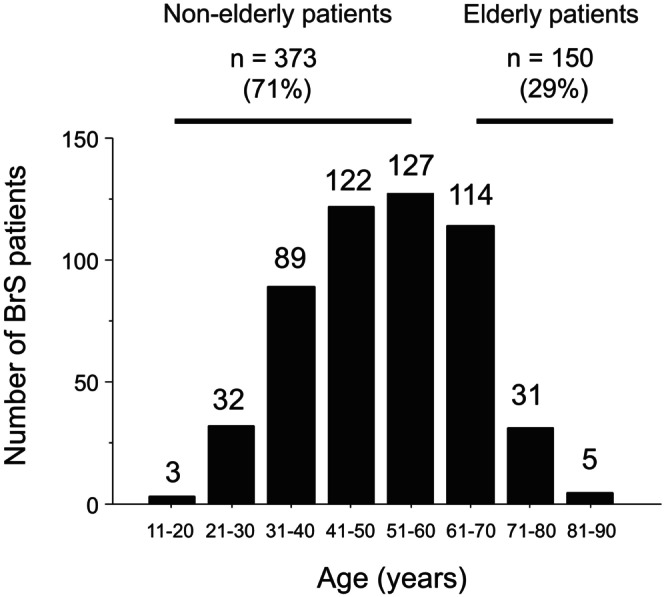
Age distribution of patients with BrS at study enrollment. There were 150 patients aged ≥61 years (elderly) and 373 patients <60 years old (nonelderly). BrS, Brugada syndrome.

In accordance with the report by Minier et al.,^5^ patients were divided into two groups according to age at enrollment: elderly BrS (>60 years, *n* = 150, 29%) and nonelderly BrS (≤60 years, *n* = 373, 71%) (Figure 1). Of all patients, 432 underwent programmed electrical stimulation (PES) using a uniform protocol. A wide QRS complex in lead *V*_2_ was defined as a QRS duration of >90 ms. The J wave in the inferolateral leads and late potentials on signal‐averaged ECG were defined as previously reported.^13^ The T‐peak‐to‐T‐end interval of ≥100 ms was defined as the maximum T‐peak‐to‐T‐end interval that measured ≥100 ms in any of the precordial leads.

This study was approved by the ethics committees of Tsukuba University (Approval number: H29‐122) and the ethics review committee of each participating institution. Written informed consent was obtained from all patients.

### 2.2 Programmed electrical stimulation

The stimulation protocol comprised two drive pacing cycles (600 and 400 ms) and was applied for up to three ventricular extra stimuli down to a minimum of 200 ms from the right ventricular apex (RVA) and right ventricular outflow tract (RVOT). The respective number of extra stimuli at both drive pacing cycles and order of pacing sites were as follows: single from the RVA, double from the RVA, single from the RVOT, double from the RVOT, triple from the RVA, and triple from the RVOT.^13^ Positive PES (PES+) was defined as induced VF or sustained polymorphic VT requiring direct current shock by PES. The decision to perform PES on patients with BrS was based on each physician's clinical judgment.

### 2.3 Clinical course

Every 12 months, the patients were monitored for clinical events and incidence of CEs, including appropriate ICD shock therapy for fast polymorphic VT or VF >200 beats/min in patients with ICD and documented fast VT, VF, or SCD in patients without ICD. The indication for ICD implantation was based on the physician's clinical judgment, according to the Japanese Circulation Society guidelines for BrS.^15^ Each CE in patients with ICD was analyzed using the stored intracardiac ECG in the ICD and was confirmed as appropriate for fast VT or VF.

### 2.4 Statistical analysis

Data were expressed as mean ± SD for continuous variables or as numbers and percentages for categorical variables. The Shapiro–Wilk test was used to determine the normal distribution of continuous variables. The Student's *t‐*test was used to compare normally distributed continuous variables, and the Mann–Whitney *U*‐test was used to compare nonnormally distributed continuous variables. Other categorical variables were examined using Fisher's exact test. Event‐rate curves were generated using the Kaplan–Meier method. Univariate and multivariate Cox proportional hazards regression analyses were performed to identify independent predictors of CE occurrence in elderly patients with BrS. For all tests, *p* values <0.05 were considered statistically significant. All statistical analyses were conducted using JMP v13.2.1 (SAS, Cary, NC, USA) on Windows™ 11 (Microsoft, Redmond, WA, USA).

## 3 RESULTS

### 3.1 Baseline clinical characteristics

Of the 523 patients, 227 (43%) had a history of symptoms, such as VF in 99 (19%) and unexplained syncope, which was presumed to be arrhythmogenic, in 128 (24%). The mean follow‐up period in all patients was 106 ± 62 months. The elderly BrS group comprised 29% of all patients; the age range was 61–70 years in 114 (22%), 71–80 years in 31 (6%), and 81–90 years in 5 (1%). As shown in Table 1, Compared with the nonelderly BrS group, the elderly BrS group had significantly higher proportions of patients with unexplained syncope (*p* = 0.03) and the presence and/or history of atrial fibrillation (*p* = 0.005); a significantly lower proportion of patients with wide QRS complex in lead *V*_2_ (53% vs. 68%, *p* = 0.003). There were no significant differences in any other factors between the elderly and nonelderly BrS groups.

**TABLE 1:** Clinical and electrophysiological characteristics of elderly and nonelderly patients.

	Elderly BrS patients *n* = 150	Nonelderly BrS patients *n* = 373	*p* value
Gender (male), *n* (%)	140 (93)	357 (96)	0.27
Age at enrollment (years)	68 ± 6	45 ± 10	<0.001**
History of unexplained syncope, *n* (%)	47 (31)	81 (22)	0.03*
History of VF, *n* (%)	22 (15)	77 (21)	0.14
History and/or presence of AF, *n* (%)	34 (23)	47 (13)	0.005**
Spontaneous type 1 ECG, *n* (%)	99 (66)	220 (59)	0.14
Family history of SCD, *n* (%)	33 (22)	92 (25)	0.57
J wave in inferolateral leads, *n* (%)	18 (12)	43 (12)	0.88
Wide QRS complex in lead *V*_2_ (>90 ms), *n* (%)	76 (53)	246 (68)	0.003**
Fragmented QRS, *n* (%)	10 (7)	13 (3)	0.15
Positive late potential, *n*/*N* (%)	77/104 (74)	218/283 (77)	0.59
PES‐induced VT/VF, *n*/*N* (%)	84/120 (70)	212/312 (68)	0.73
ICD implantation, *n* (%)	98 (67)	238 (64)	0.61
Follow‐up periods (months)	108 ± 61	105 ± 63	0.65

*Note*: Values are presented as mean ± SD or *n* (%).

Abbreviations: AF, atrial fibrillation; ECG, electrocardiogram; ICD, implantable cardioverter‐defibrillator; PES, programmed electrical stimulation; SCD, sudden cardiac death; VF, ventricular fibrillation; VT, ventricular tachycardia.

* *p* < 0.05.

** *p* < 0.01.

### 3.2 Clinical differences in the incidence of life‐threatening arrhythmias between elderly and nonelderly patients with BrS

**FIGURE 2:**
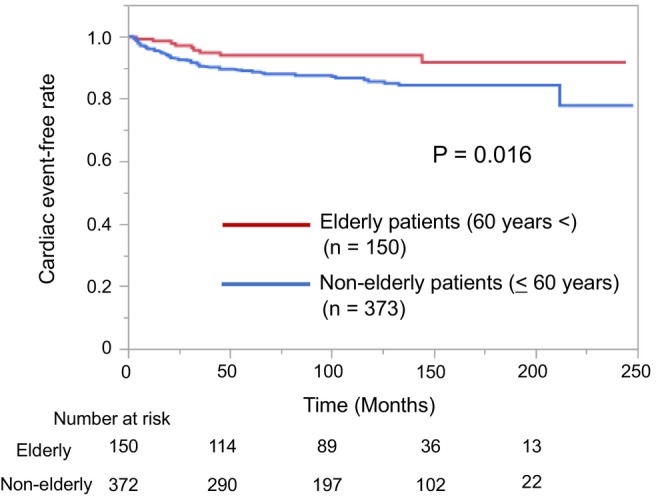
Kaplan–Meier analysis of cardiac event‐free survival in elderly and nonelderly patients with BrS. On follow‐up, the cardiac event‐free rate is significantly higher in the elderly BrS group than in the nonelderly BrS group (*p* = 0.016). BrS, Brugada syndrome.

Overall, 59 (11%) patients experienced CEs during follow‐up. In particular, CEs occurred in 9 (6%) patients in the elderly BrS group and 50 (13%) patients in the nonelderly BrS group, with annual incidences of 0.7% and 1.5%, respectively. Kaplan–Meier analysis showed that the incidence of CEs was significantly lower in the elderly BrS group than in the nonelderly BrS group (*p* = 0.016, Figure 2). Of the 215 nonelderly asymptomatic BrS patients, 9 patients had CEs, and only 2 patients developed CEs when they were over 60 years (at the ages of 64 and 69).

**FIGURE 3:**
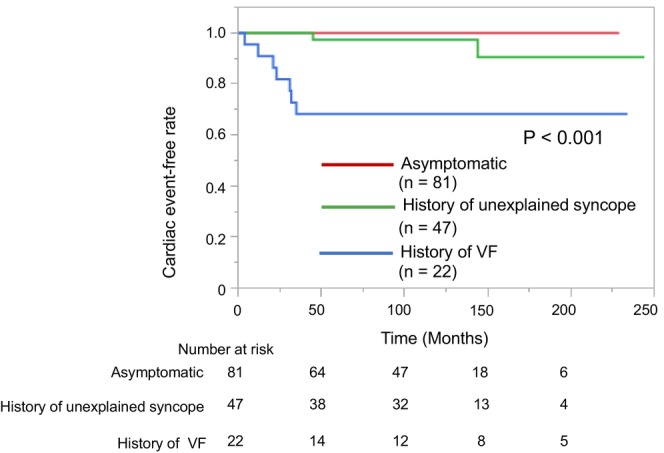
Kaplan–Meier analysis of cardiac event‐free survival according to symptoms among elderly patients with BrS. On follow‐up, the cardiac event‐free rate among elderly patients is significantly lower in those with a history of VF than in those with unexplained syncope and who were asymptomatic (*p* < 0.001). VF, ventricular fibrillation.

**FIGURE 4:**
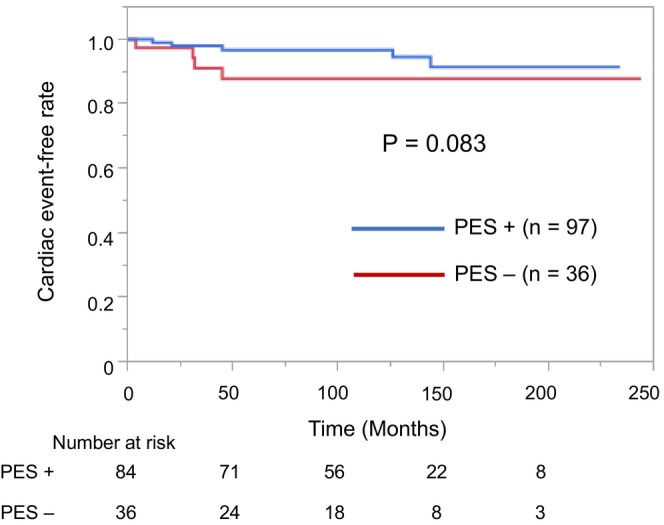
Kaplan–Meier analysis of cardiac event‐free survival in the PES+ and PES− groups. The cardiac event‐free rate is not significantly different between the PES+ and PES− groups (*p* = 0.083). PES, programmed electrical stimulation.

The clinical and electrophysiological characteristics of elderly patients with BrS according to CEs are presented in Table 2. The proportion of patients with a history of VF was significantly higher in patients with CEs than in those without CEs (*p* < 0.001), but there was no significant difference in the history of unexplained syncope (*p* = 0.72). Furthermore, the annual incidence of CEs was higher in patients with a history of VF (3.7%) than in those with unexplained syncope (0.5%) and those who were asymptomatic (0%). All patients with CEs had undergone ICD implantation and received appropriate ICD shocks for VF. The proportion of PES+ patients was not significantly different between patients with and without CEs (43% vs. 72%, respectively, *p* = 0.19; Table 2). Among elderly patients with BrS, Kaplan–Meier analysis revealed that the incidence of CEs was significantly higher in those with a history of VF compared with other factors (*p* < 0.001) (Figure 3). However, the annual incidence of CEs in those with a history of unexplained syncope was relatively low and did not differ significantly from that in asymptomatic patients (0.5% vs. 0%, respectively, *p* = 0.56). Figure 4 shows that there was no significant difference in the CE‐free rate between the PES+ and PES− groups in elderly patients with BrS (*p* = 0.083).

**TABLE 2:** Clinical and electrophysiological characteristics of elderly patients with BrS according to the absence and presence of cardiac events.

	Cardiac event (−) *n* = 141	Cardiac event (+) *n* = 9	*p* value
Gender (male), *n* (%)	131 (93)	9 (100)	1.00
Age at enrollment (years)	68 ± 6	68 ± 7	0.91
History of unexplained syncope, *n* (%)	45 (32)	2 (22)	0.72
History of VF, *n* (%)	15 (11)	7 (78)	<0.001**
History and/or presence of AF, *n* (%)	30 (21)	4 (44)	0.12
Spontaneous type 1 ECG, *n* (%)	91 (65)	8 (89)	0.27
Family history of SCD, *n* (%)	30 (21)	3 (33)	0.41
J wave in inferolateral leads, *n* (%)	17 (12)	1 (11)	1.00
Wide QRS complex in lead *V*_2_ (>90 ms), *n* (%)	70 (52)	6 (67)	0.50
Fragmented QRS, *n* (%)	9 (6)	1 (11)	0.47
T‐peak‐to‐T‐end interval (≥100 ms), *n* (%)	69 (49)	4 (44)	0.79
Positive late potential, *n*/*N* (%)	72/99 (73)	5/5 (100)	0.32
PES‐induced VT/VF, *n*/*N* (%)	81/113 (72)	3/7 (43)	0.19
ICD implantation, *n* (%)	89 (64)	9 (100)	0.03*
Follow‐up periods (months)	112 ± 59	39 ± 41	<0.001**

*Note*: Values are presented as mean ± SD or *n* (%).

Abbreviations: AF, atrial fibrillation; ECG, electrocardiogram; ICD, implantable cardioverter‐defibrillator; PES, programmed electrical stimulation; SCD, sudden cardiac death; VF, ventricular fibrillation; VT, ventricular tachycardia.

* *p* < 0.05.

** *p* < 0.001.

### 3.3 Univariate and multivariate analyses of factors associated with CE occurrence

As shown in Table 3, among elderly patients with BrS, a history of VF was found to be significantly associated with the occurrence of CEs on univariate analysis (*p* < 0.001) and independently predicted the occurrence of CEs on multivariate logistic regression analysis (hazard ratio 23.5, 95% confidence interval 5.13–175; *p* < 0.001).

**TABLE 3:** Univariate and multivariate analyses of factors associated with the occurrence of cardiac events.

Parameters	HR	95% CI	*p* value	Multivariate HR	95%CI	*p* value
Male	n.a	n.a	0.23	—	—	—
Age at enrollment (years)	1.03	0.90–1.14	0.68	1.06	0.94–1.17	0.32
Family history of SCD	1.78	0.37–6.76	0.44	—	—	—
History of unexplained syncope	2.18	0.56–14.3	0.28	—	—	—
History of VF	22.0	5.30–148	<0.001**	23.5	5.13–175	<0.001**
Spontaneous type 1 ECG	4.23	0.78–78.5	0.10	2.41	0.21–28.2	0.75
QRS duration in lead *V*_2_ (>90 ms)	1.83	0.48–8.68	0.38	—	—	—
Presence of J wave in inferior and/or lateral leads	1.18	0.22–22.0	0.87	—	—	—
PES‐induced VT/VF	0.29	0.06–1.31	0.10	—	—	—
Fragmented QRS	1.66	0.11–11.2	0.66	—	—	—
T‐peak‐to‐T‐end interval (≥100 ms)	1.19	0.31–4.65	0.79	—	—	—
Positive late potential	n.a	n.a	0.07	—	—	—

Abbreviations: AF, atrial fibrillation; ECG, electrocardiogram; n.a, not available; RVA, right ventricular apex; RVOT, right ventricular outflow tract; SCD, sudden cardiac death; VF, ventricular fibrillation; VT, ventricular tachycardia.

** *p* < 0.001.

### 3.4 Clinical and electrophysiological characteristics of elderly patients with BrS with CEs

The clinical and electrophysiological characteristics of the nine elderly patients with BrS with CEs are presented in Table 4. Three patients were aged >70 years at the time of CE occurrence (case 3, 76 years; case 8, 73 years; and case 9, 87 years). All patients in this subgroup had a history of symptoms, such as VF (*n* = 7) and unexplained syncope (*n* = 2). The positive and negative predictive values for the history of symptoms were 13% and 100%, respectively. All patients had undergone ICD implantation. Eight patients (89%) had spontaneous type 1 ECG.

**TABLE 4:** Clinical and electrophysiological characteristics of elderly patients with BrS and cardiac events (*n* = 9).

	Case 1	Case 2	Case 3	Case 4	Case 5	Case 6	Case 7	Case 8	Case 9
Gender (male)	+	+	+	+	+	+	+	+	+
Age at enrollment (years)	61	63	64	65	66	67	69	70	85
Age at cardiac event (years)	64	67	76	68	67	69	69	73	87
History of symptom (VF or syncope)	+ (VF)	+ (syncope)	+ (syncope)	+ (VF)	+ (VF)	+ (VF)	+ (VF)	+ (VF)	+ (VF)
History of AF	+	−	−	+	−	−	+	+	−
Spontaneous type 1 ECG	−	+	+	+	+	+	+	+	+
Family history of SCD	−	−	−	−	+	+	−	+	−
Presence of J wave in inferolateral leads	−	+	−	−	−	−	−	−	−
Wide QRS complex in lead *V*_2_ (>90 ms)	+	+	+	+	−	−	+	+	−
Fragmented QRS	−	−	−	−	−	−	+	−	−
Positive late potential	Positive	n.a	Positive	Positive	n.a	n.a	Positive	Positive	n.a
PES‐induced VT/VF	n.a	−	+	−	+	n.a	−	−	+
ICD implantation	+	+	+	+	+	+	+	+	+
Follow‐up periods at cardiac event (months)	35	45	144	31	12	23	4	32	21

Abbreviations: AF, atrial fibrillation; ECG, electrocardiogram; ICD, implantable cardioverter‐defibrillator; n.a, not available; PES, programmed electrical stimulation; SCD, sudden cardiac death; VF, ventricular fibrillation; VT, ventricular tachycardia.

## 4 DISCUSSION

### 4.1 Main findings

This study had three main findings. First, compared with nonelderly patients, elderly patients with BrS had a significantly lower incidence of CEs. Second, the history of VF was associated with and was an independent predictor of CE occurrence in elderly patients with BrS. Third, the absence of symptoms, such as VF and/or syncope, was associated with a high negative predictive value for CEs. None of the elderly patients with BrS who were asymptomatic at enrollment experienced CE during follow‐up.

Among elderly patients with BrS, life‐threatening arrhythmias associated with BrS are unlikely, especially when they are asymptomatic, and a history of VF can be useful for risk stratification for the occurrence of CEs. To the best of our knowledge, this was the first multicenter prospective study to examine the incidence of life‐threatening arrhythmias and risk‐stratified elderly patients with BrS.

### 4.2 Incidence of life‐threatening arrhythmias in elderly patients with BrS

Similar to previous reports,^7^, ^16^ this study found that elderly patients with BrS had a lower incidence of CEs compared with nonelderly patients. As suggested by Conte et al.,^4^ this finding is probably the result of various physical changes associated with aging. Although we cannot explain the detailed mechanism of the decreased risk of life‐threatening arrhythmias with aging, we propose several possibilities. First, age‐related sex hormone changes may be involved. High testosterone levels are known to play an important role in ECG waveform patterns and male preponderance among patients with BrS.^11^ According to Ezaki et al.,^12^ neoadjuvant androgen deprivation therapy for prostate cancer significantly reduced the J point height. Considering that testosterone, which can increase outward potassium currents and decrease inward calcium currents, decreases with aging,^17^ sex hormone changes may explain the lower incidence of CEs in elderly patients with BrS. The second possibility is that changes in cardiac autonomic nervous system function may play a role. In patients with BrS, fatal arrhythmias are more likely to occur during nighttime sleep, and vagus nerve activity has been implicated in this phenomenon.^18^ In general, autonomic nervous system activity decreases with age.^19^ This may explain why CE is less likely to occur in elderly patients with BrS.

### 4.3 Risk factors for life‐threatening arrhythmias among elderly patients with BrS

In elderly patients with BrS, the incidence of CEs significantly differed among patients with a history of VF, those with a history of unexplained syncope, and those who were asymptomatic. Consistent with the results of previous large clinical trials,^2^ we found that a history of VF was the main risk factor for the occurrence of CEs. Conversely, the incidence of CEs in elderly BrS patients with a history of unexplained syncope was low and not significantly different from that in asymptomatic patients. Notably, the 0.5% annual incidence of CE among patients with a history of syncope in this population was lower than that those previously reported (1.7% and 1.9%).^2^, ^20^ The incidence of syncope generally increases with aging, with a sharp rise after the age of 70 years.^21^ We speculate that this may be because the elderly are more prone to syncope secondary to other causes, such as ischemic heart disease and cerebral infarction, rather than BrS‐related syncope. A pooled analysis conducted in 2016 reported that the risk of VF occurrence could be high if ≤2 consecutive stimuli are applied for VF induction.^22^ Moreover, the current Japanese guidelines state that patients with BrS who have unexplained syncope should undergo PES and receive ICD implantation if VF is induced by ≤2 consecutive stimuli (Class IIa).^15^ Our results suggest that the risk of developing CEs in elderly BrS patients with unexplained syncope is very low, and this should be considered.

In this study, three patients experienced CEs at the age of >70 years. Kamakura et al. reported that in a single‐center study, only two patients aged >70 years developed VF, which was recurrent and complicated by ischemic heart disease.^7^ In the elderly, the risk of developing VF due to ischemic heart disease is generally high. Therefore, it may be possible that different VF triggers (such as myocardial ischemia) are involved in the occurrence of VF in elderly patients with BrS than in nonelderly patients. However, because we did not have data on the presence or absence of myocardial ischemia at the time CE onset in the three patients, we cannot rule out the possibility that it was not caused by BrS. Further studies are required to clarify the relationship between aging and the incidence of life‐threatening arrhythmias in patients with BrS.

Because of aging, it may be expected that the prolongation and fragmentation of QRS are more common in the elderly than in the young. In this study, however, the proportion of patients with conduction abnormalities (wide QRS complex) was significantly lower in elderly patients with BrS than in nonelderly patients. We speculate that we enrolled elderly patients less affected by conduction disturbance due to aging, which means the enrolled elderly patients did not have severe conduction abnormality.

### 4.4 Role of PES in the risk stratification of patients with BrS

Previous studies have shown conflicting results on the prognostic value of PES in patients with BrS.^23^, ^24^ Several studies have indicated that PES has a high negative predictive value as a tool for risk stratification.^22^, ^25^ However, the usefulness of PES for the predictive value in elderly patients with BrS has not been fully evaluated. To the best of our knowledge, this study using PES is the largest survey on patients with BrS in Japan and the only multicenter study that included a large number of elderly patients with BrS. This study revealed that VT/VF induction on PES did not have a predictive value for the occurrence of CEs in elderly patients with BrS. In this study, all nine elderly patients who developed CE had a history of VF or unexplained syncope. However, four patients who were negative PES (PES−) had CE during follow‐up. These results suggest that the indications for ICD therapy, including replacement, in elderly patients with BrS should be based on symptoms rather than VT/VF induction by PES.

### 4.5 Clinical implications

The risk of developing life‐threatening arrhythmia in patients with BrS decreases with age. However, the risk stratification of elderly patients with BrS has not been studied in detail. This study showed that the risk of developing CEs decreases after the age of 60, and that the risk may decrease even further in asymptomatic patients. However, the risk should not be ignored in elderly patients with symptomatic BrS. It is important to evaluate the indication for an ICD in elderly patients with BrS because elderly patients are more likely to have various problems associated with ICD implantation and replacement. Our results suggest that new ICD implantation or replacement surgery should be considered carefully in elderly patients without VF history.

### 4.6 Limitations

This study had several limitations. First, the number of patients aged >60 years at enrollment was relatively small, and only nine elderly patients experienced a CE during follow‐up. Second, aging and other factors may have led to changes in the ECG findings during the disease course, although this study was based on ECG findings at the time of enrollment. Further detailed investigation, including ECG changes, is needed. Third, genetic test results were not registered in the J‐IVFS database and, therefore, could not be evaluated. Addition of genetic analysis may allow more accurate risk stratification. Fourth, this study examined patients with BrS who were older. This means that patients who had CEs at a younger age were excluded, and it cannot be denied that there is a bias.

## 5 CONCLUSIONS

Elderly patients with BrS have a better prognosis than nonelderly patients. Moreover, VF history was an independent predictor of the occurrence of life‐threatening arrhythmias, even in elderly patients with BrS. Our results suggest that asymptomatic elderly patients with BrS may be at very low risk of developing life‐threatening arrhythmias.

## FUNDING INFORMATION

This research did not receive any specific grant from any funding agency in the public, commercial, or not‐for‐profit sectors.

## CONFLICT OF INTEREST STATEMENT

The authors declare that they have no conflicts of interest.

## PATIENT CONSENT STATEMENT

Written informed consent was obtained from all patients.

## Data Availability

The data underlying this article will be shared on reasonable request to the corresponding author.
